# Supplementary material for: Pathophysiological Significance of Neutrophilic Transfer RNA-Derived Small RNAs in Asymptomatic Moyamoya Disease
Source: Cells. 2021 May 1;10(5):1086. doi: 10.3390/cells10051086 (PMC8147334; doi:10.3390/cells10051086)

2D Structure

```
111                              143
                        8mer-1a
5'-aaGACTTATAACAGGACTTTTACATATGTGGGa-3' utr
     ||  |:  ||||  |    :|   ||  :|||||||
3'-ucCUUAGGAUUGGC---GAUCUG-GUACACCCu-5' tRF
              16    151413    7 6 5 4 3 2
             3' Pairing          Seed
```

Local AU

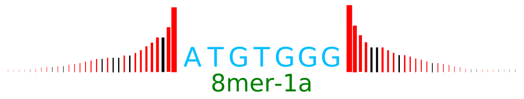

Position

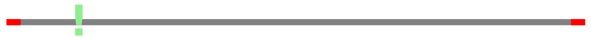

Supplement: Supplementary file 1 [file cells-10-01086-s001.zip › cells-1149161 3rd supplementary materials/Data Supplements/Data Supplement V.zip!/Data supplement 6-mRNAs targets of tsRNAs/tsRNA-Glu-TTC-010/Diagrams/tRF-Glu-TTC-010_vs_ENST00000039989(TTC17).pdf]
